# Higher Total Cholesterol Concentration May Be Associated with Better Cognitive Performance among Elderly Females

**DOI:** 10.3390/nu14194198

**Published:** 2022-10-09

**Authors:** Ke Pang, Chunxia Liu, Jianbin Tong, Wen Ouyang, Shuntong Hu, Yongzhong Tang

**Affiliations:** 1 Department of Anesthesiology, Third Xiangya Hospital, Central South University, Changsha 410013, China; 2 Department of Neurology, Third Xiangya Hospital, Central South University, Changsha 410013, China

**Keywords:** cognitive performance, elderly, female, total cholesterol, NHANES

## Abstract

Background: The brain contains the highest level of cholesterol in the body, and the total amount of serum cholesterol in the blood has a huge impact on brain aging and cognitive performance. However, the association of total serum cholesterol with cognitive function remains uncertain. This study determines whether there is an association between the total amount of cholesterol in the blood and cognitive performance in elderly females without a history of stroke. Methods: This population-based cross-sectional study was conducted on elderly (over 60 years old) females and males without a history of stroke from 2011 to 2014 in the US National Health and Nutrition Examination Survey (NHANES). The primary exposure was total blood cholesterol, and the main outcome was cognitive performance; this association was assessed with logistic regression analysis and restricted cubic splines. Results: 1309 female and 1272 male participants were included. In females, higher total cholesterol was significantly associated with higher cognitive scores, particularly in the digit symbol substitution test (OR 0.51, 95% CI (0.36–0.72)) and the animal fluency test (OR 0.64, 95% CI (0.45–0.91)). This association remained significant in models adjusted for age, race, smoking status, education level, and chronic conditions (OR 0.40, 95% CI (0.25–0.63)). This association was not significant in males, however. Conclusions: A higher concentration of total cholesterol measured in later life may be a protective factor for cognitive performance among females over 60 years old without a history of stroke. Further, this association was more pronounced among women with higher levels of education than women with lower or no education.

## 1. Introduction

Dementia, or Alzheimer’s disease, is the fifth leading cause of death for individuals 65 years of age and older in the United States [1]. Worldwide, mild cognitive impairment affects 10–15% of the population aged 60 years or more [2]. Cognitive impairment results in various symptoms, including memory disorders, language decline, and execution ability disorder [3,4]. Cognitive impairments have become a key challenge for public health [5].

It has been hypothesized that lipid metabolism is associated with cardiovascular health problems [6]. However, lipid metabolism also plays an important role in cognitive health [7]. Docosahexaenoic acid (DHA) is a structural constituent of membranes in the development of the central nervous system and potentially influences cognitive decline in adult life [8]. The brain contains the highest level of cholesterol in the body [9], and cholesterol is important for brain activity. For example, the myelin, which provides an insulating layer for neurons to increase the brain’s processing speed, contains 46% cholesterol [10]. Cholesterol is also associated with dopamine transport as it is present in the crystal structures of dopamine transporters (DATs) [11,12]. Meanwhile, low cholesterol levels are associated with a decrease in the number of serotonin receptors, leading to an overall reduction in serotonergic transmission in the brain [13]. Another related study revealed that a reduced turnover of serotonergic compounds in the brain, namely 5-HT, is associated with cognitive decline, including impaired long-term memory function and cognitive flexibility [14]. Besides the two informational molecules in brain, the decline in some hormones—including estrogen, progesterone, DHEA, and testosterone, whose precursors, namely pregnenolone, were independent of cholesterol—is associated with some types of mental syndrome or low cognitive performance [15,16,17,18].

However, in clinical practice, the relationship between total cholesterol and cognitive performance [19], and the dose–response association between total cholesterol and cognitive performance, remain uncertain. Some researchers believe that this relationship may be age-related [20]. A high total cholesterol measured in midlife is associated with a higher probability of aging-related dementia and late-life cognitive impairment [21,22,23,24,25,26,27,28]; this risk could be mitigated if cholesterol is found to be a protective factor in late life [29,30].

Thus, we analyzed a sample of older females over 60 years old in the National Health and Nutrition Examination Survey (NHANES) to elucidate the association between total cholesterol and cognition in late life.

## 2. Methods

### 2.1. Study Population

The National Health and Nutrition Examination Survey (NHANES) is a survey research program conducted to assess the health and nutritional status of adults and children in the United States, tracking changes over time. It began in 1971 and became an annual event in 1999. In each cycle, approximately 5000 representative people are selected via a complex process of multistage probability sampling. All participants complete an informed consent document. The survey combines interviews, physical examinations, and laboratory tests. The participants first take part in an interview at home; physical examination and laboratory tests are conducted in the mobile examination center (MEC) before a call-back consultation is carried out within the next few days.

**Figure 1:**
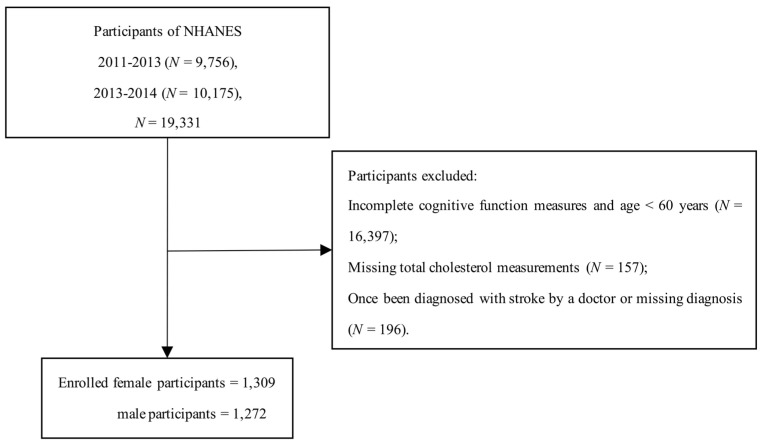
Flow chart outlining the process for selecting participants.

Two cycles (2011–2012 and 2013–2014) of data with the most recent information on cognitive function measures were collected and combined for our research. A total of 19,931 individuals participated in the NHANES from 2011 to 2014. A total of 2934 of the participants aged 60 or older were used in our research because only this sample of participants received a cognitive assessment. Among these participants, we further excluded those without total cholesterol measurements (*N* = 157) and participants with a history of stroke (*N* = 197). Next, the study enrolled 1309 female and 1272 male participants aged 60 or older (Figure 1).

### 2.2. Cholesterol Measurement

An enzymatic assay method and the Trinder reaction (Roche Modular P chemistry analyzer) were used to measure total cholesterol. A heparin–manganese precipitation method or a direct immunoassay technique was used to measure high-density lipoprotein cholesterol [31,32,33,34].

### 2.3. Cognitive Performance Assessment

The cognitive function of older participants (aged 60 or over) was estimated using four tests [35,36]: (1) the Consortium to Establish a Registry for Alzheimer’s Disease (CERAD) test, (2) the animal fluency test, (3) the digit symbol substitution test (DSST), and (4) the Consortium to Establish a Registry for Alzheimer’s Disease (CERAD) delayed recall test. The four tests are widely used in cohort studies to estimate memory, language, speech, and cognitive functions and to screen risk factors [37,38,39].

The CERAD [40] includes a word list recall test (immediate and delayed). The immediate word list recall measures mild cognitive impairment; the participant is asked to recall stimuli immediately after they are presented. This is used to identify mild dementia. The word list delayed recall is a verbal declarative memory test using a visually or verbally presented word list where participants are asked to remember as many words as possible after a specified delay interval in order to quantify memory performance [41,42].

The AFT is widely used to estimate the cognitive abilities of patients with various neurological diseases. The test requires participants to name as many animals as possible in a short period of time. A low AFT score reflects an impairment of language function [43,44]. It has been proven to identify patients with mild cognition impairment, dementia, and normal aging [45,46].

The DSST consisted of digit–symbol pairs followed by a list of digits. The participants aim to write the corresponding symbol from the 133 boxes that hold adjacent numbers as quickly as possible. The number of correct symbols within the allowed time (2 min) is the score [47].

Due to the ceiling and floor effect caused by a wide range of cognitive function in the elderly population, namely, the scale attenuation effect [48,49], we created a global cognitive score by averaging the standardized scores of the three cognitive tests scores (the CERAD, animal fluency, and Digit symbol substitution tests) [50,51]. As there are no standard cutoff points for the CERAD, animal fluency, and DSST tests to identify low or normal cognitive function, we used the 25th percentile of the scores among different groups of participants as the cutoff point, which followed the method used in previous studies [52]. Given the significant effect of age on cognitive function, the global score was stratified at different ages (60–69 years, 70–79 years, and ≥80 years) [53]. For each test or global score, the participants were divided into a low-cognitive-performance group and a normal-cognitive-performance group—the former group’s scores were less than or equal to the corresponding cutoff values, while the latter group’s scores were higher than the corresponding cutoff values.

### 2.4. Covariates

A variety of covariates were introduced into our study according to previous research [53,54]; these covariates are considered to have an association with cognitive function decline: race (Mexican American, other Hispanic, non-Hispanic White, non-Hispanic Black, or other race), age (60–70, 70–80, and ≥80 years), education level (less than 9th grade, 9–11th grade, above 11th grade), poverty–income ratio (<1 and ≥1), smoking (smoked less than 100 cigarettes in life, smoked more than 100 cigarettes in life but does not smoke now, smoked more than 100 cigarettes and still smokes), body mass index (BMI) (normal: <25 kg/m^2^; overweight: 25 to 30 kg/m^2^; obese: ≥30 kg/m^2^), marital status, drinking (having at least 12 alcohol drinks per year or not), diabetes (was diagnosed with diabetes or the value of fasting glycated hemoglobin more than 6.4 [55]), and chronic conditions including hypertension and stroke.

### 2.5. Statistical Analysis

Statistical analysis was performed on the original datasets extracted from the NHANES and a complex sample analysis was performed using the stratification, cluster, and sample weight variables provides in the NHANES [56]. A new sample weight variable for the two combined datasets from 2011 to 2014 was created by taking one twice for the 2-year weight for each participant sampled from 2011 to 2012 and one twice for the 2-year weight for each person sampled from 2013 to 2014. We used R 4.1.0 programming for statistical analysis and the R survey package to appropriately weigh analyses for the complex, multistage sampling design of NHANES. Continuous variable data were expressed as mean ± standard deviation, and categorical variables were expressed as percentages. Pearson’s chi-square or Fisher’s exact tests were used to compare the categorical variables between low- and normal-cognitive-performance groups. A Student’s *t*-test or Mann–Whitney U-test was used to compare different levels of continuous variables between groups. A two-sided *p* < 0.05 was considered statistically significant.

In this study, the total cholesterol values among female and male participants were categorized into four groups according to first quartile, medium, and third quartile. For the main outcome (low or normal cognitive performance), single-variable logistic regression was performed to analyze the association between cognitive performance and total cholesterol in an unadjusted model. Multivariable logistic regression was performed and controlled for the effects of the following covariates of cognitive function: age, education level, race, smoking and drinking status, poverty–income ratio, BMI, chronic conditions (including diabetes and hypertension), and marriage status. The variables including age, race, education level, marriage status, poverty–income ratio, and BMI were used for adjustment in Model 2; the variables, including Model 2 plus drinking status, smoking status, and chronic conditions (hypertension and diabetes), were used for adjustment in Model 3.

## 3. Results

The demographic characteristics of all the participants (*N* = 2581) and of the female (*N* = 1309) and the male (*N* = 1272) participants in the final study sample were summarized and shown in Table 1, Table 2 and Table 3, respectively. The results showed that the decline in cognitive performance was significantly associated with race, ratio of family income to poverty, chronic conditions (including diabetes and hypertension), drinking status, education level, and total cholesterol (*p* < 0.01). Participants who were considered to have a cognitive impairment were more likely to be Mexican American, other Hispanic, non-Hispanic Black, and other races; have lower educational level and poverty–income ratio; have chronic conditions (including diabetes and hypertension); drink more; be widowed, divorced, separated, or never married; or have lower total cholesterol levels. For DSST, the prevalence of smoking (including currently smoking or previously smoked) in participants with low cognitive performance was significantly higher than that of people with normal cognitive performance. However, a higher concentration of total cholesterol was significantly associated with cognitive function only among older female participants; in older male participants, this association was not significant. 

Table 4 shows the relationship between the different dimensions of cognitive performance (including the animal fluency test, the digit symbol substitution test, and the CERAD test) and the different total cholesterol levels among older female participants.

In Model 1, compared with the low total cholesterol measurement, those reporting 201–229 mg/dL of total cholesterol had an odds ratio (OR) with a 95% confidence interval (CI) of 0.72 (95% CI, 0.52–1.00) for AST score, 0.53 (95% CI, 0.37–0.75) for DSST score, and 0.49 (95% CI,0.34–0.69) for global cognitive performance; those reporting more than 229 mg/dL had an odds ratio (OR) with a 95% confidence interval (CI) of 0.51 (95% CI, 0.36–0.72) for AST score, 0.64 (95% CI, 0.45–0.91) for DSST score, 0.69 (95% CI, 0.48–0.97) for CERAD test, and 0.41 (95% CI, 0.28–0.59) for global cognitive performance.

After adjustments for age, race, education level, the ratio of family income to poverty line, marriage status, and BMI, 201–229 mg/dL of total cholesterol was associated with the DSST (OR = 0.56 (95% CI, 0.35–0.87)) and global cognitive performance (OR = 0.50 (95% CI, 0.33–0.77)), whereas more than 229 mg/dL of total cholesterol was associated with different dimensions of cognitive performance (0.52 (95% CI, 0.35–0.77) for AST score, 0.70 (95% CI, 0.48–1.04) for CERAD score, and 0.34 (95% CI, 0.22–0.54) for global cognitive performance).

Additionally, in Model 3, compared with low total cholesterol levels, those who had 201–229 mg/dL of total cholesterol had a multivariate-adjusted OR (95% CI) of 0.62 (95% CI, 0.39–0.99) for DSST, and 0.58 (95% CI, 0.37–0.90) for global cognitive performance, and those who had more than 229 mg/dL of total cholesterol had a multivariate-adjusted OR (95% CI) of 0.57 (95% CI, 0.38–0.85) for AST score, and 0.40 (95% CI, 0.25–0.63) for global cognitive performance. The trend test shown in Table 4 showed that cognitive performance (including global cognitive performance and the AST score) was associated with total cholesterol in a dose-dependent manner (*p*_trend_ < 0.05) in an adjusted model, and that a higher dose of total cholesterol had a greater advantage for the protection of cognition.

However, Table 5 showed that, among older female participants, the association between total cholesterol and cognitive function was not obvious before or after adjustment for other variables in the logistic regression analysis. We performed a sub-groups analysis in terms of education level, as shown in Table 6. The result showed that higher total cholesterol was significantly associated with normal cognitive performance in people who have received higher education, while higher total cholesterol was normally associated with normal cognitive performance in people with a medium level of education. 

Restricted cubic spline analyses between total cholesterol and the Animal Fluency Test score (A), the DDST (B), the CERAD test (C), and the global cognitive performance (D). The solid line and dashed lines represent the estimated ORs and their 95% confidence intervals (OR, odds ratio).

**Figure 2:**
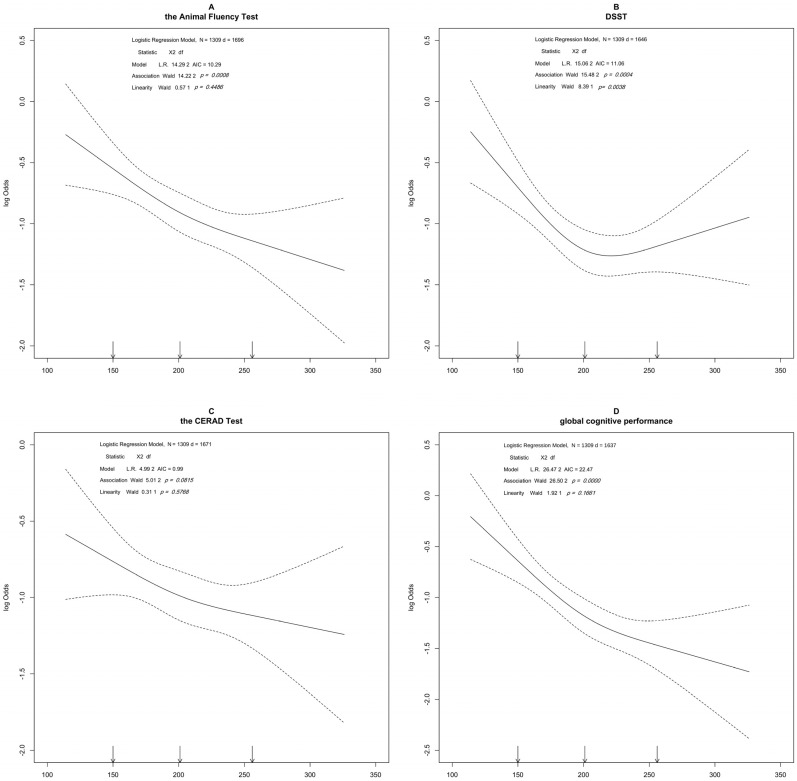
Restricted cubic spline analyses for female participants. Restricted cubic spline analyses between total cholesterol and the Animal Fluency Test score (**A**), the DDST (**B**), the CERAD test (**C**) and the global cognitive performance (**D**). The solid line and dashed line represent the estimated ORs and their 95% confidence intervals (OR, odds ratio).

Figure 2 described the results of the restricted cubic spline analyses between the Animal Fluency test, the DSST, the CERAD test, and the global cognitive performance, respectively, among female participants. The prevalence of impaired cognitive function decreased with the increasing value of total cholesterol and showed a potential nonlinear association.

The baseline characteristics of the approximately 25 million persons represented by the 1309 female participants are shown in Supplementary Table S1, in which the result was similar to that in Table 2. Additionally, we found similar results (Supplementary Table S2) that total cholesterol was a protective factor for cognitive performance with logistic regression, especially in the Animal Fluency Test, the Digit Symbol Substitution Test, and global cognitive performance. Furthermore, the trend analysis in Supplementary Table S2 suggested a dose-dependent protective role for total cholesterol in cognitive performance. Additionally, the missing data in the original dataset of the female participants are shown in Supplementary Table S3.

## 4. Discussion

With the increase in life expectancy, cognitive impairment is an important public health problem; furthermore, the incidence of cognitive impairment is increasing. A systematic review has reported that the global prevalence of cognitive impairment ranged from 5.1% to 41% [57]. The factors leading to cognitive decline vary, including increasing age, lower education, race, and chronic conditions such as stroke and diabetes [58]. However, it has not been confirmed whether the amount of total cholesterol measured in late life is a protective factor for cognitive function.

In this study, we combined data including NHANES 2011–2012 and 2013–2014. This research explored data provided by 1309 female Americans aged 60 years or older who had no history of stroke. The 1309 female participants are representative of approximately twenty-five million people in America. In the unadjusted model, a high concentration of total cholesterol was statistically associated with higher global cognitive and animal fluency test scores. In the model adjusted for age, race, education levels, income–poverty ratio, history of diabetes, and smoking status, total cholesterol remained associated with global cognitive and animal fluency scores.

To date, evidence for the relationship between total cholesterol and cognitive function in elderly populations has been equivocal and inconclusive, likely due to the effects of aging. Our findings on the relation between total cholesterol and cognition were consistent with some previous studies. Research based on 382 people studied the relationship between concentrations of total cholesterol and cognitive performance in participants ranging from 70 to 90 years old, revealing that a lower total cholesterol value is associated with poorer cognition in both non-dementia and dementia cases [59]. Another 70-year follow-up study including 1034 people showed that higher total cholesterol and HDL-C values were associated with higher cognitive scores among participants aged 70 [60], although the relationship between cholesterol levels and cognition were mostly no longer significant after considering IQ test scores recorded in early life.

There have also been studies revealing total cholesterol’s harmful effects on cognition. A study found that higher blood concentrations of total cholesterol were related to faster global cognitive decline in a population of 1159 Chinese adults aged over 60 [61]; the associations between all lipids and cognitive decline appeared to be more significant among individuals over 100 years old. Another study found that higher circulating total cholesterol indicated an increased risk of mild cognitive impairment in elderly females [62]. However, these two studies enrolled participants who were different from the participants in our studies, and the cognitive scale they used was different from the scales used in our study [63]. The population enrolled in the former study investigated Chinese adults over 60 years who had only received a basic education [64]. However, our sub-groups analysis found that higher total cholesterol was only significantly associated with normal cognitive performance among the female population who had received a higher education. Moreover, our sample size was larger, with 1309 female individuals without a history of stroke; the weighted sample size was approximately twenty-five million people. A meta-analysis based on eight studies and over 21,000 individuals aged over 60 years found that there were no relationships between cholesterol and cognitive decline or dementia in older adult groups [65].

Some studies have suggested that this relationship could be age-dependent, i.e., the higher concentration of total cholesterol measured at midlife was a risk factor for late-life cognition [66], while the higher concentration of total cholesterol measured at late-life was a risk factor for late-life cognition [19,20,21,29,30,67].

Although genetic susceptibility and metabolic function determine an individual’s lipid profile to a great extent, factors related to lifestyle may affect the clearance of cholesterol. Different kinds of diet may affect cognitive function [68,69]. In addition to dietary intake, smoking status [70], education level [71], family income [72], race, and chronic conditions were proven to be associated with cognition decline. As a result, we introduced these features as co-variables. Dietary cholesterol has been considered to increase the risk of cardiovascular disease and, for cardiovascular events, a low cholesterol level is advisable. A study reported that increased cholesterol levels were associated with a high CVD risk among young adults [73]. However, recently some studies found that total cholesterol had protective qualities for some non-cardiovascular conditions and hemorrhagic stroke [74,75], especially among the old-aged [76]. Higher total cholesterol was associated with a decrease in mortality among people over 85 years old. In addition, people who are old with a low concentration of total cholesterol had higher all-cause mortality [77]. As a result, we believe a higher concentration of serum total cholesterol signifies better health conditions in late life, in terms of both cognition performance and other non-cardiovascular and hemorrhagic stroke conditions.

## 5. Limitations

There are several limitations and strengths in our study. First, we chose the NHANES as our data source, which provided a sufficiently large sample size. Second, we used four standard scores of four cognitive tests to create a global compound cognitive score to minimize the ceiling and floor effects. Third, considering that age was the major risk factor for cognitive impairment, age was used to divide the population and to calculate the cut-off values (the lowest quartile) of each group. However, as a cross-sectional study, this research could not reveal the relationship between total cholesterol and cognitive performance for the population at different ages. Further longitudinal studies need to be conducted to investigate the nonlinear longitudinal relationship between total cholesterol and neuropsychological function and cognitive performance.

Investigating the relationship between total cholesterol and cognitive function can provide information that is significant for the prevention of cognition decline. Because this effect could be age-related, we might consider adopting different strategies in different ages in future work.

## 6. Conclusions

In America, a higher concentration of total cholesterol measured in late life may be a protective factor for cognitive performance among females over 60 years without a history of stroke. This association was more significant among females with higher education levels and was not as apparent in females with lower education levels or without an education. We postulate that a better total cholesterol concentration could be 200 mg/dL in elderly females without a medical history of strokes.

## Figures and Tables

**Table 1:** Characteristics of the study population.

	Overall	Female	Male
N	2581	1309	1272
Age in years at screening (mean (SD))	69.29 (6.74)	69.33 (6.71)	69.24 (6.78)
Age (%)			
60–70 years	1432 (55.5)	722 (55.2)	710 (55.8)
70–80 years	746 (28.9)	387 (29.6)	359 (28.2)
≥80 years	403 (15.6)	200 (15.3)	203 (16.0)
Race (%)			
Mexican American	236 (9.1)	110 (8.4)	126 (9.9)
Other Hispanic	269 (10.4)	141 (10.8)	128 (10.1)
Non-Hispanic White	1247 (48.3)	660 (50.4)	587 (46.1)
Non-Hispanic Black	580 (22.5)	279 (21.3)	301 (23.7)
Other Race	249 (9.6)	119 (9.1)	130 (10.2)
Ratio of family income to poverty (mean (SD))	2.65 (1.61)	2.53 (1.60)	2.77 (1.61)
Poverty–income ratio ≥ 1 (%)	1981 (83.8)	973 (81.0)	1008 (86.6)
Body Mass Index (mean (SD))	29.05 (6.28)	29.52 (6.91)	28.56 (5.53)
Body mass index (%)			
<25 kg/m^2^	680 (26.7)	355 (27.4)	325 (25.9)
25–30 kg/m^2^	917 (35.9)	399 (30.8)	518 (41.3)
≥30 kg/m^2^	954 (37.4)	542 (41.8)	412 (32.8)
Diabetes (%)	694 (26.9)	329 (25.2)	365 (28.7)
Had at least 12 alcohol drinks/year (%)	1746 (68.8)	707 (54.7)	1039 (83.4)
Hypertension (%)	1570 (60.9)	834 (63.8)	736 (58.0)
Marital status (%)			
Widowed/divorced/separated/never married	1067 (41.3)	705 (53.8)	362 (28.4)
Married/living with partner	1514 (58.7)	604 (46.2)	910 (71.6)
Smoking status (%)			
Never	1295 (50.2)	819 (62.6)	476 (37.5)
Former	962 (37.3)	356 (27.2)	606 (47.7)
Current	322 (12.5)	133 (10.2)	189 (14.9)
Educational level (%)			
Below high school	634 (24.6)	319 (24.4)	315 (24.8)
High school	606 (23.5)	318 (24.3)	288 (22.7)
Above high school	1339 (51.9)	672 (51.3)	667 (52.5)
Total Cholesterol (mean (SD))	192.24 (43.16)	202.88 (41.71)	181.28 (41.89)
Statin drugs used (%)	1071 (41.5)	502 (38.3)	569 (44.7)

Data show number of subjects (percentage) or medians (interquartile ranges).

**Table 2:** Characteristics of the female population (*N* = 1309).

	The Animal Fluency Test			The Digit Symbol Substitution Test			The CERAD Test			The Global Performance		
	Normal Cognitive Performance	Low Cognitive Performance	*p* Value	Normal Cognitive Performance	Low Cognitive Performance	*p* Value	Normal Cognitive Performance	Low Cognitive Performance	*p* Value	Normal Cognitive Performance	Low Cognitive Performance	*p* Value
N (%)	922.00 (70.44)	387.00 (29.56)		972.00 (74.26)	337.00 (25.74)		947.00 (72.35)	362 (27.65)		981 (74.94)	328 (25.06)	
Age in years at screening ^2^	69.22 (6.70)	69.58 (6.74)	0.3	69.17 (6.79)	69.77 (6.45)	0.083	69.05 (6.75)	70.06 (6.56)	0.009	69.12 (6.80)	69.94 (6.41)	0.024
Age (%) ^1^			0.4			>0.9			0.5			>0.9
60–70 years	508.00 (55.10)	214.00 (55.30)		534.00 (54.94)	188.00 (55.79)		530.00 (55.97)	192.00 (53.04)		541.00 (55.15)	181.00 (55.18)	
70–80 years	280.00 (30.37)	107.00 (27.65)		290.00 (29.84)	97.00 (28.78)		272.00 (28.72)	115.00 (31.77)		290.00 (29.56)	97.00 (29.57)	
≥80 years	134.00 (14.53)	66.00 (17.05)		148.00 (15.23)	52.00 (15.43)		145.00 (15.31)	55.00 (15.19)		150.00 (15.29)	50.00 (15.24)	
Race (%) ^1^			<0.001			<0.001			<0.001			<0.001
Mexican American	75.00 (8.13)	35.00 (9.04)		54.00 (5.56)	56.00 (16.62)		69.00 (7.29)	41.00 (11.33)		64.00 (6.52)	46.00 (14.02)	
Other Hispanic	82.00 (8.89)	59.00 (15.25)		74.00 (7.61)	67.00 (19.88)		80.00 (8.45)	61.00 (16.85)		77.00 (7.85)	64.00 (19.51)	
Non-Hispanic White	536.00 (58.13)	124.00 (32.04)		583.00 (59.98)	77.00 (22.85)		507.00 (53.54)	153.00 (42.27)		571.00 (58.21)	89.00 (27.13)	
Non-Hispanic Black	157.00 (17.03)	122.00 (31.52)		170.00 (17.49)	109.00 (32.34)		203.00 (21.44)	76.00 (20.99)		178.00 (18.14)	101.00 (30.79)	
Other Race	72.00 (7.81)	47.00 (12.14)		91.00 (9.36)	28.00 (8.31)		88.00 (9.29)	31.00 (8.56)		91.00 (9.28)	28.00 (8.54)	
Ratio of family income to poverty ^2^	2.72 (1.62)	2.05 (1.45)	<0.001	2.85 (1.59)	1.58 (1.20)	<0.001	2.72 (1.60)	2.03 (1.48)	<0.001	2.78 (1.59)	1.76 (1.39)	<0.001
Poverty–income ratio (%) ^1^			<0.001			<0.001			<0.001			<0.001
≤0.99	129.00 (15.09)	99.00 (28.61)		108.00 (12.03)	120.00 (39.60)		126.00 (14.53)	102.00 (30.54)		116.00 (12.87)	112.00 (37.33)	
≥1	726.00 (84.91)	247.00 (71.39)		790.00 (87.97)	183.00 (60.40)		741.00 (85.47)	232.00 (69.46)		785.00 (87.13)	188.00 (62.67)	
Body mass index (%) ^1^			0.2			0.6			0.7			0.7
<25 kg/m^2^	252.00 (27.45)	103.00 (27.25)		261.00 (26.91)	94.00 (28.83)		255.00 (27.13)	100.00 (28.09)		272.00 (27.93)	83.00 (25.78)	
25–30 kg/m^2^	295.00 (32.14)	104.00 (27.51)		306.00 (31.55)	93.00 (28.53)		296.00 (31.49)	103.00 (28.93)		301.00 (30.90)	98.00 (30.43)	
≥30 kg/m^2^	371.00 (40.41)	171.00 (45.24)		403.00 (41.55)	139.00 (42.64)		389.00 (41.38)	153.00 (42.98)		401.00 (41.17)	141.00 (43.79)	
Body Mass Index (kg/m^2^) ^2^	29.33 (6.74)	29.99 (7.29)	0.2	29.35 (6.78)	30.03 (7.25)	0.2	29.50 (6.98)	29.59 (6.73)	0.7	29.32 (6.86)	30.12 (7.02)	0.079
Diabetes (%)	198.00 (21.48)	131.00 (33.94)	<0.001	208.00 (21.40)	121.00 (36.01)	<0.001	212.00 (22.41)	117.00 (32.32)	<0.001	201.00 (20.51)	128.00 (39.02)	<0.001
Had at least 12 alcohol drinks/year (%) ^1^	520.00 (57.21)	187.00 (48.83)	0.006	576.00 (59.75)	131.00 (39.94)	<0.001	534.00 (57.05)	173.00 (48.60)	0.006	568.00 (58.44)	139.00 (43.44)	<0.001
Hypertension (%) ^1^	558.00 (60.59)	276.00 (71.50)	<0.001	593.00 (61.13)	241.00 (71.51)	<0.001	593.00 (61.13)	241.00 (71.51)	0.3	598.00 (61.02)	236.00 (72.17)	<0.001
Marital status (%) ^1^			0.036			<0.001			0.012			0.009
Widowed/divorced/separated/never married	479.00 (51.95)	225.00 (58.29)		494.00 (50.88)	210.00 (62.31)		489.00 (51.69)	215.00 (59.39)		507.00 (51.73)	197.00 (60.06)	
Married/living with partner	443.00 (48.05)	161.00 (41.71)		477.00 (49.12)	127.00 (37.69)		457.00 (48.31)	147.00 (40.61)		473.00 (48.27)	131.00 (39.94)	
Smoking status (%) ^1^			0.2			0.005			0.4			0.2
Never	562.00 (61.02)	257.00 (66.41)		587.00 (60.45)	232.00 (68.84)		591.00 (62.47)	228.00 (62.98)		604.00 (61.63)	215.00 (65.55)	
Former	262.00 (28.45)	94.00 (24.29)		287.00 (29.56)	69.00 (20.47)		264.00 (27.91)	92.00 (25.41)		280.00 (28.57)	76.00 (23.17)	
Current	97.00 (10.53)	36.00 (9.30)		97.00 (9.99)	36.00 (10.68)		91.00 (9.62)	42.00 (11.60)		96.00 (9.80)	37.00 (11.28)	
Educational level (%) ^1^			<0.001			<0.001			<0.001			<0.001
Below high school	169.00 (18.33)	150.00 (38.76)		127.00 (13.07)	192.00 (56.97)		172.00 (18.16)	147.00 (40.61)		144.00 (14.68)	175.00 (53.35)	
High school	219.00 (23.75)	99.00 (25.58)		243.00 (25.00)	75.00 (22.26)		228.00 (24.08)	90.00 (24.86)		235.00 (23.96)	83.00 (25.30)	
Above high school	534.00 (57.92)	138.00 (35.66)		602.00 (61.93)	70.00 (20.77)		547.00 (57.76)	125.00 (34.53)		602.00 (61.37)	70.00 (21.34)	
Total Cholesterol (mg/dL) ^2^	205.62 (41.43)	196.36 (41.71)	<0.001	204.66 (40.52)	197.75 (44.63)	0.003	204.41 (41.82)	198.88 (41.22)	0.039	206.13 (41.15)	193.17 (41.94)	<0.001
Total Cholesterol (%) ^1^			0.002			0.003			0.14			<0.001
<174 mg/dL	208.00 (22.56)	120.00 (31.01)		221.00 (22.74)	107.00 (31.75)		227.00 (23.97)	101.00 (27.90)		215.00 (21.92)	113.00 (34.45)	
174–201 mg/dL	232.00 (25.16)	96.00 (24.81)		242.00 (24.90)	86.00 (25.52)		230.00 (24.29)	98.00 (27.07)		238.00 (24.26)	90.00 (27.44)	
201–229 mg/dL	235.00 (25.49)	98.00 (25.32)		265.00 (27.26)	68.00 (20.18)		245.00 (25.87)	88.00 (24.31)		265.00 (27.01)	68.00 (20.73)	
>229 mg/dL	247.00 (26.79)	73.00 (18.86)		244.00 (25.10)	76.00 (22.55)		245.00 (25.87)	75.00 (20.72)		263.00 (26.81)	57.00 (17.38)	
Statin drugs used (%) ^1^	337.00 (36.55)	165.00 (42.64)	0.039	362.00 (37.24)	140.00 (41.54)	0.2	351.00 (37.06)	151.00 (41.71)	0.12	353.00 (35.98)	149.00 (45.43)	0.002

Data show number of subjects (percentage) or medians (interquartile ranges); ^1^ Chi-square test was used to compare the percentage between participants with and without low cognitive performance; ^2^ Wilcoxon rank sum or Fisher’s exact tests were used to compare the mean ± standard deviance values between participants with and without low cognitive performance.

**Table 3:** Characteristics of the male population (*N* = 1272).

	The Animal Fluency Test			The Digit Symbol Substitution Test			The CERAD Test			The Global Performance		
	Normal Cognitive Performance	Low Cognitive Performance	*p* Value	Normal Cognitive Performance	Low Cognitive Performance	*p* Value	Normal Cognitive Performance	Low Cognitive Performance	*p* Value	Normal Cognitive Performance	Low Cognitive Performance	*p* Value
N (%)	893 (70.20)	379 (29.80)		939 (73.82)	333 (26.18)		918 (72.17)	354 (27.83)		952 (74.84)	320 (25.16)	
Age in years at screening ^2^	69.28 (6.73)	69.16 (6.91)	0.7	69.16 (6.78)	69.48 (6.81)	0.5	69.06 (6.78)	69.73 (6.78)	0.12	69.17 (6.74)	69.45 (6.91)	0.6
Age (%) ^1^			0.4			>0.9			0.4			>0.9
60–70 years	491.00 (54.98%)	219.00 (57.78%)		525.00 (55.91%)	185.00 (55.56%)		522.00 (56.86%)	188.00 (53.11%)		531.00 (55.78%)	179.00 (55.94%)	
70–80 years	262.00 (29.34%)	97.00 (25.59%)		265.00 (28.22%)	94.00 (28.23%)		250.00 (27.23%)	109.00 (30.79%)		269.00 (28.26%)	90.00 (28.12%)	
≥80 years	140.00 (15.68%)	63.00 (16.62%)		149.00 (15.87%)	54.00 (16.22%)		146.00 (15.90%)	57.00 (16.10%)		152.00 (15.97%)	51.00 (15.94%)	
Race (%) ^1^			<0.001			<0.001			<0.001			<0.001
Mexican American	92.00 (10.30%)	34.00 (8.97%)		79.00 (8.41%)	47.00 (14.11%)		80.00 (8.71%)	46.00 (12.99%)		85.00 (8.93%)	41.00 (12.81%)	
Other Hispanic	85.00 (9.52%)	43.00 (11.35%)		61.00 (6.50%)	67.00 (20.12%)		80.00 (8.71%)	48.00 (13.56%)		75.00 (7.88%)	53.00 (16.56%)	
Non-Hispanic White	470.00 (52.63%)	117.00 (30.87%)		511.00 (54.42%)	76.00 (22.82%)		456.00 (49.67%)	131.00 (37.01%)		504.00 (52.94%)	83.00 (25.94%)	
Non-Hispanic Black	173.00 (19.37%)	128.00 (33.77%)		175.00 (18.64%)	126.00 (37.84%)		206.00 (22.44%)	95.00 (26.84%)		188.00 (19.75%)	113.00 (35.31%)	
Other Race	73.00 (8.17%)	57.00 (15.04%)		113.00 (12.03%)	17.00 (5.11%)		96.00 (10.46%)	34.00 (9.60%)		100.00 (10.50%)	30.00 (9.38%)	
Ratio of family income to poverty ^2^	2.93 (1.62)	2.40 (1.52)	<0.001	3.10 (1.58)	1.85 (1.30)	<0.001	2.92 (1.61)	2.38 (1.55)	<0.001	3.01 (1.59)	2.04 (1.43)	<0.001
Poverty-income ratio (%) ^1^			0.07			<0.001			0.008			<0.001
≤0.99	101.00 (12.24%)	55.00 (16.22%)		80.00 (9.27%)	76.00 (25.25%)		100.00 (11.79%)	56.00 (17.72%)		95.00 (10.80%)	61.00 (21.48%)	
≥1	724.00 (87.76%)	284.00 (83.78%)		783.00 (90.73%)	225.00 (74.75%)		748.00 (88.21%)	260.00 (82.28%)		785.00 (89.20%)	223.00 (78.52%)	
Body mass index (kg/m^2^) ^2^	28.74 (5.43)	28.13 (5.74)	0.02	28.66 (5.51)	28.28 (5.58)	0.3	28.65 (5.41)	28.32 (5.83)	0.2	28.66 (5.42)	28.28 (5.83)	0.2
Body Mass Index (%) ^1^			0.2			0.8			0.7			0.8
<25 kg/m^2^	217.00 (24.63%)	108.00 (28.88%)		236.00 (25.46%)	89.00 (27.13%)		230.00 (25.39%)	95.00 (27.22%)		242.00 (25.77%)	83.00 (26.27%)	
25–30 kg/m^2^	362.00 (41.09%)	156.00 (41.71%)		383.00 (41.32%)	135.00 (41.16%)		374.00 (41.28%)	144.00 (41.26%)		384.00 (40.89%)	134.00 (42.41%)	
≥30kg/m^2^	302.00 (34.28%)	110.00 (29.41%)		308.00 (33.23%)	104.00 (31.71%)		302.00 (33.33%)	110.00 (31.52%)		313.00 (33.33%)	99.00 (31.33%)	
Diabetes (%) ^1^	236.00 (26.43%)	129.00 (34.04%)	0.006	235.00 (25.03%)	130.00 (39.04%)	<0.001	257.00 (28.00%)	108.00 (30.51%)	0.4	251.00 (26.37%)	114.00 (35.62%)	0.002
Had at least 12 alcohol drinks/year (%) ^1^	748.00 (84.90%)	291.00 (79.73%)	0.025	783.00 (84.65%)	256.00 (79.75%)	0.042	765.00 (84.62%)	274.00 (80.12%)	0.056	794.00 (84.65%)	245.00 (79.55%)	0.037
Hypertension (%) ^1^	508.00 (56.95%)	228.00 (60.32%)	0.3	529.00 (56.46%)	207.00 (62.16%)	0.07	532.00 (58.02%)	204.00 (57.79%)	>0.9	545.00 (57.31%)	191.00 (59.87%)	0.4
Marriage status (%) ^1^			0.2			<0.001			0.15			<0.001
Widowed/divorced/separated/never married	244.00 (27.35%)	117.00 (30.87%)		231.00 (24.63%)	130.00 (39.04%)		250.00 (27.26%)	111.00 (31.36%)		244.00 (25.66%)	117.00 (36.56%)	
Married/living with partner	648.00 (72.65%)	262.00 (69.13%)		707.00 (75.37%)	203.00 (60.96%)		667.00 (72.74%)	243.00 (68.64%)		707.00 (74.34%)	203.00 (63.44%)	
Smoking status (%) ^1^			0.093			<0.001			0.007			0.001
Never	339.00 (38.00%)	137.00 (36.15%)		370.00 (39.40%)	106.00 (31.93%)		329.00 (35.88%)	147.00 (41.53%)		365.00 (38.38%)	111.00 (34.69%)	
Former	433.00 (48.54%)	173.00 (45.65%)		459.00 (48.88%)	147.00 (44.28%)		462.00 (50.38%)	144.00 (40.68%)		465.00 (48.90%)	141.00 (44.06%)	
Current	120.00 (13.45%)	69.00 (18.21%)		110.00 (11.71%)	79.00 (23.80%)		126.00 (13.74%)	63.00 (17.80%)		121.00 (12.72%)	68.00 (21.25%)	
Educational Level (%) ^1^			<0.001			<0.001			<0.001			<0.001
Below high school	173.00 (19.37%)	142.00 (37.67%)		123.00 (13.10%)	192.00 (58.01%)		176.00 (19.19%)	139.00 (39.38%)		150.00 (15.76%)	165.00 (51.89%)	
High school	191.00 (21.39%)	97.00 (25.73%)		214.00 (22.79%)	74.00 (22.36%)		209.00 (22.79%)	79.00 (22.38%)		219.00 (23.00%)	69.00 (21.70%)	
Above high school	529.00 (59.24%)	138.00 (36.60%)		602.00 (64.11%)	65.00 (19.64%)		532.00 (58.02%)	135.00 (38.24%)		583.00 (61.24%)	84.00 (26.42%)	
Total Cholesterol (mg/dL) ^2^	182.95 (42.69)	177.36 (39.72)	0.081	181.53 (41.96)	180.58 (41.74)	>0.9	180.91 (42.42)	182.25 (40.52)	0.3	181.00 (42.08)	182.12 (41.37)	0.4
Total Cholesterol (%) ^1^			0.4			0.7			0.4			0.2
<151 mg/dL	216.00 (24.19%)	107.00 (28.23%)		235.00 (25.03%)	88.00 (26.43%)		240.00 (26.14%)	83.00 (23.45%)		251.00 (26.37%)	72.00 (22.50%)	
151–178 mg/dL	224.00 (25.08%)	95.00 (25.07%)		242.00 (25.77%)	77.00 (23.12%)		227.00 (24.73%)	92.00 (25.99%)		229.00 (24.05%)	90.00 (28.12%)	
178–208 mg/dL	226.00 (25.31%)	92.00 (24.27%)		237.00 (25.24%)	81.00 (24.32%)		236.00 (25.71%)	82.00 (23.16%)		245.00 (25.74%)	73.00 (22.81%)	
>208 mg/dL	227.00 (25.42%)	85.00 (22.43%)		225.00 (23.96%)	87.00 (26.13%)		215.00 (23.42%)	97.00 (27.40%)		227.00 (23.84%)	85.00 (26.56%)	
Statin drugs used (%) ^1^	406.00 (45.46%)	163.00 (43.01%)	0.4	426.00 (45.37%)	143.00 (42.94%)	0.4	417.00 (45.42%)	152.00 (42.94%)	0.4	441.00 (46.32%)	128.00 (40.00%)	0.049

Data show number of subjects (percentage) or medians (interquartile ranges); ^1^ Chi-square test was used to compare the percentage between participants with and without low cognitive performance; ^2^ Wilcoxon rank sum test or Fisher’s exact test was used to compare the mean ± standard deviance values between participants with and without low cognitive performance.

**Table 4:** Odds ratios (95% confidence intervals) for four dimensions of cognitive performance across quartiles of total cholesterol among female participants, NHANES 2011–2014 (*N* = 1309).

Animal Fluency Test				DSST			CERAD Test			Global Cognitive Performance		
	Model 1	Model 2	Model 3	Model 1	Model 2	Model 3	Model 1	Model 2	Model 3	Model 1	Model 2	Model 3
0–174	1.00 (Ref.)	1.00 (Ref.)	1.00 (Ref.)	1.00 (Ref.)	1.00 (Ref.)	1.00 (Ref.)	1.00 (Ref.)	1.00 (Ref.)	1.00 (Ref.)	1.00 (Ref.)	1.00 (Ref.)	1.00 (Ref.)
174–201	0.72 * (0.52–0.99)	0.76 (0.53–1.10)	0.82 (0.56–1.20)	0.73 (0.52–1.03)	0.77 (0.50–1.19)	0.83 (0.53–1.29)	0.96 (0.69–1.34)	1.10 (0.76–1.58)	1.20 (0.83–1.76)	0.72 (0.52–1.00)	0.85 (0.57–1.26)	0.94 (0.62–1.42)
201–229	0.72 (0.52–1.00)	0.80 (0.55–1.16)	0.88 (0.59–1.29)	0.53 *** (0.37–0.75)	0.56 * (0.35–0.87)	0.62 * (0.39–0.99)	0.81 (0.58–1.13)	0.86 (0.59–1.25)	0.95 (0.64–1.41)	0.49 *** (0.34–0.69)	0.50 ** (0.33–0.77)	0.58 * (0.37–0.90)
>229	0.51 *** (0.36–0.72)	0.52 ** (0.35–0.77)	0.57 ** (0.38–0.85)	0.64 * (0.45–0.91)	0.72 (0.46–1.13)	0.79 (0.50–1.26)	0.69 * (0.48–0.97)	0.70 (0.48–1.04)	0.78 (0.52–1.16)	0.41 *** (0.28–0.59)	0.34 *** (0.22–0.54)	0.40 *** (0.25–0.63)
*p*_trend_	<0.001	<0.003	0.015	0.003	0.061	0.177	0.022	0.042	0.134	<0.001	<0.001	<0.001

Calculated using binary logistic regression; Reference (Ref.); Model 2 adjusted for age, education level, body mass index (BMI), marriage status, ratio of family income to poverty and race; Model 3 adjusted for age and race, educational level, marriage status, ratio of family income to poverty, body mass index (BMI), drinking status, smoking status, hypertension, and diabetes. * *p* < 0.05; ** *p* < 0.01; *** *p* < 0.001.

**Table 5:** Odds ratios (95% confidence intervals) for four dimensions of cognitive performance across quartiles of total cholesterol among male participants, NHANES 2011–2014 (*N* = 1272).

Animal Fluency Test				DSST			CERAD Test			Global Cognitive Performance		
	Model 1	Model 2	Model 3	Model 1	Model 2	Model 3	Model 1	Model 2	Model 3	Model 1	Model 2	Model 3
0–151	1.00 (Ref.)	1.00 (Ref.)	1.00 (Ref.)	1.00 (Ref.)	1.00 (Ref.)	1.00 (Ref.)	1.00 (Ref.)	1.00 (Ref.)	1.00 (Ref.)	1.00 (Ref.)	1.00 (Ref.)	1.00 (Ref.)
151–178	0.86 (0.61–1.19)	0.78 (0.54–1.14)	0.80 (0.54–1.18)	0.85 (0.60–1.21)	0.97 (0.62–1.53)	1.16 (0.72–1.85)	1.17 (0.83–1.66)	1.10 (0.76–1.61)	1.16 (0.78–1.72)	1.37 (0.96–1.96)	1.45 (0.95–2.22)	1.51 (0.97–2.34)
178–208	0.82 (0.59–1.15)	0.75 (0.51–1.08)	0.79 (0.53–1.17)	0.91 (0.64–1.30)	0.96 (0.61–1.51)	1.25 (0.77–2.03)	1.00 (0.70–1.43)	0.91 (0.61–1.34)	0.93 (0.61–1.40)	1.04 (0.72–1.50)	0.97 (0.63–1.50)	1.09 (0.69–1.74)
>208	0.76 (0.54–1.06)	0.70 (0.47–1.02)	0.76 (0.51–1.15)	1.03 (0.73–1.46)	0.99 (0.63–1.56)	1.26 (0.78–2.06)	1.30 (0.92–1.85)	1.26 (0.86–1.85)	1.30 (0.86–1.96)	1.31 (0.91–1.88)	1.23 (0.80–1.90)	1.36 (0.86–2.16)
*p*_trend_	0.107	0.063	0.206	0.777	0.943	0.325	0.246	0.433	0.392	0.377	0.734	0.416

Calculated using binary logistic regression; Reference (Ref.); Model 2 adjusted for age, education level, body mass index (BMI), marriage status, ratio of family income to poverty, and race; Model 3 adjusted for age and race, educational level, marriage status, ratio of family income to poverty, body mass index (BMI), drinking status, smoking status, hypertension, and diabetes.

**Table 6:** Odds ratios (95% confidence intervals) for four dimensions of cognitive performance across quartiles of total cholesterol in different education sub-groups among female participants.

		Animal Fluency Test			DSST			CERAD Test			Global Cognitive Performance		
		Model 1	Model 2	Model 3	Model 1	Model 2	Model 3	Model 1	Model 2	Model 3	Model 1	Model 2	Model 3
Less than 9th grade	0–174	1.00 (Ref.)	1.00 (Ref.)	1.00 (Ref.)	1.00 (Ref.)	1.00 (Ref.)	1.00 (Ref.)	1.00 (Ref.)	1.00 (Ref.)	1.00 (Ref.)	1.00 (Ref.)	1.00 (Ref.)	1.00 (Ref.)
	174–201	0.54 (0.29–1.01)	0.49 * (0.24–0.99)	0.58 (0.28–1.19)	0.78 (0.42–1.46)	0.57 (0.26–1.24)	0.62 (0.27–1.41)	1.42 (0.77–2.63)	1.43 (0.72–2.85)	1.57 (0.76–3.28)	0.93 (0.51–1.73)	0.98 (0.49–2.00)	1.08 (0.51–2.31)
	201–229	1.21 (0.66–2.25)	1.43 (0.71–2.91)	1.60 (0.76–3.42)	0.69 (0.37–1.30)	0.68 (0.30–1.53)	0.66 (0.28–1.58)	1.59 (0.86–2.95)	1.59 (0.78–3.29)	1.66 (0.77–3.59)	1.05 (0.57–1.95)	1.36 (0.65–2.91)	1.56 (0.71–3.51)
	>229	0.54 * (0.29–0.98)	0.63 (0.32–1.24)	0.64 (0.31–1.30)	0.76 (0.41–1.40)	0.74 (0.34–1.62)	0.84 (0.37–1.89)	0.93 (0.51–1.70)	1.06 (0.53–2.11)	1.24 (0.60–2.57)	0.60 (0.33–1.10)	0.54 (0.26–1.09)	0.63- (0.30–1.31)
9–11th grade	0–174	1.00 (Ref.)	1.00 (Ref.)	1.00 (Ref.)	1.00 (Ref.)	1.00 (Ref.)	1.00 (Ref.)	1.00 (Ref.)	1.00 (Ref.)	1.00 (Ref.)	1.00 (Ref.)	1.00 (Ref.)	1.00 (Ref.)
	174–201	1.11 (0.60–2.08)	1.02 (0.52–2.01)	1.14 (0.56–2.33)	0.75 (0.38–1.48)	0.70 (0.32–1.50)	0.71 (0.32–1.58)	1.22 (0.64–2.35)	1.24 (0.63–2.45)	1.46 (0.72–2.99)	0.83 (0.44–1.57)	0.86 (0.43–1.71)	1.06 (0.52–2.20)
	201–229	0.61 (0.30–1.21)	0.51 (0.23–1.10)	0.60 (0.26–1.36)	0.51 (0.23–1.07)	0.40 * (0.16–0.96)	0.49 (0.19–1.20)	0.93 (0.46–1.86)	0.90 (0.42–1.91)	1.11 (0.50–2.46)	0.35 ** (0.16–0.73)	0.27 ** (0.11–0.64)	0.35 * (0.13–0.83)
	>229	0.75 (0.37–1.53)	0.53 (0.22–1.21)	0.60 (0.24–1.44)	1.00 (0.48–2.07)	0.88 (0.37–2.05)	1.10 (0.45–2.63)	0.86 (0.40–1.79)	0.69 (0.29–1.58)	0.83 (0.34–1.95)	0.59 (0.28–1.22)	0.44 (0.18–1.02)	0.54 (0.22–1.30)
above 11th grade	0–174	1.00 (Ref.)	1.00 (Ref.)	1.00 (Ref.)	1.00 (Ref.)	1.00 (Ref.)	1.00 (Ref.)	1.00 (Ref.)	1.00 (Ref.)	1.00 (Ref.)	1.00 (Ref.)	1.00 (Ref.)	1.00 (Ref.)
	174–201	0.74 (0.44–1.26)	0.71 (0.39–1.26)	0.68 (0.37–1.23)	1.09 (0.57–2.11)	0.98 (0.46–2.10)	1.06 (0.48–2.34)	0.74 (0.43–1.27)	0.72 (0.40–1.27)	0.78 (0.44–1.40)	0.68 (0.37–1.26)	0.56 (0.28–1.11)	0.59 (0.29–1.18)
	201–229	0.72 (0.43–1.20)	0.73 (0.41–1.29)	0.78 (0.43–1.42)	0.60 (0.29–1.22)	0.67 (0.30–1.46)	0.74 (0.33–1.67)	0.58 * (0.33–0.99)	0.55 * (0.31–0.98)	0.61 (0.33–1.11)	0.37 ** (0.18–0.71)	0.33 ** (0.15–0.68)	0.37 * (0.16–0.78)
	>229	0.47 ** (0.27–0.81)	0.47 * (0.25–0.86)	0.47 * (0.25–0.90)	0.52 (0.24–1.09)	0.63 (0.28–1.43)	0.65 (0.28–1.53)	0.57 * (0.32–0.98)	0.52 * (0.29–0.93)	0.57 (0.31–1.04)	0.18 *** (0.07–0.40)	0.14 *** (0.05–0.35)	0.15 *** (0.05–0.38)

Calculated using binary logistic regression; Reference (Ref.); Model 2 adjusted for age, education level, body mass index (BMI), marriage status, ratio of family income to poverty and race; Model 3 adjusted for age and race, educational level, marriage status, ratio of family income to poverty, body mass index (BMI), drinking status, smoking status, hypertension, and diabetes. * *p* < 0.05; ** *p* < 0.01; *** *p* < 0.001.

## Data Availability

Restrictions apply to the availability of these data. Data were obtained from NHANES and are available at https://wwwn.cdc.gov/nchs/nhanes/Default.aspx (accessed on 26 March 2022) [78].

## Supplementary Materials

The following supporting information can be downloaded at: https://www.mdpi.com/article/10.3390/nu14194198/s1, Table S1: Characteristics of the study population after weighting; Table S2: Weighted odds ratios (95% confidence intervals) for four dimensions of cognitive performance across quartiles of total cholesterol in survey data; Table S3: Missing Data of Variables for Table 2.
