# Supplementary material for: Higher Total Cholesterol Concentration May Be Associated with Better Cognitive Performance among Elderly Females
Source: Nutrients. 2022 Oct 9;14(19):4198. doi: 10.3390/nu14194198 (PMC9571708; doi:10.3390/nu14194198)
Supplement: Supplementary file 1 [file nutrients-14-04198-s001.zip › Supplement Table S1.pdf]

Supplement Table S1 Characteristics of the study population after weighting

	Animal Fluency			Digit Symbol Substitution Test			CERAD			Global Performance		
**Cognitive Performance**	Normal	Low	*P* value	Normal	Low	*P* value	Normal	Low	*P* value	Normal	Low	*P* value
**Number of subjects (%)**[1]	20320377.00 (79.27%)	5313175.00 (20.73%)		21740932.00 (84.81%)	3892620.00 (15.19%)		19615677.00 (76.52%)	6017875.00 (23.48%)		21437873.00 (83.63%)	4195679.00 (16.37%)	
**Age in years at screening** [2]	68.88 (6.61)	70.84 (6.71)	<0.001	68.96 (6.63)	71.13 (6.62)	<0.001	68.89 (6.63)	70.58 (6.66)	0.003	68.92 (6.66)	71.17 (6.45)	<0.001
**Age (%)** [1]			0.001			0.002			0.026			0.007
60–70 years	11761769.53 (57.88%)	2450781.33 (46.13%)		12416592.67 (57.11%)	1795958.19 (46.14%)		11339238.31 (57.81%)	2873312.55 (47.75%)		12247011.11 (57.13%)	1965539.75 (46.85%)	
70–80 years	5852504.39 (28.80%)	1719377.99 (32.36%)		6301163.33 (28.98%)	1270719.06 (32.64%)		5419408.26 (27.63%)	2152474.12 (35.77%)		6207114.92 (28.95%)	1364767.46 (32.53%)	
≥80 years	2706102.80 (13.32%)	1143015.84 (21.51%)		3023176.02 (13.91%)	825942.62 (21.22%)		2857030.54 (14.57%)	992088.09 (16.49%)		2983747.16 (13.92%)	865371.47 (20.63%)	
**Race (%)** [1]			<0.001			<0.001			0.005			<0.001
Mexican American	555433.71 (2.73%)	274866.06 (5.17%)		415900.19 (1.91%)	414399.58 (10.65%)		522313.64 (2.66%)	307986.14 (5.12%)		484040.62 (2.26%)	346259.15 (8.25%)	
Other Hispanic	593292.15 (2.92%)	415813.53 (7.83%)		521746.44 (2.40%)	487359.25 (12.52%)		573835.95 (2.93%)	435269.74 (7.23%)		542337.31 (2.53%)	466768.37 (11.12%)	
Non-Hispanic White	17191287.56 (84.60%)	3284767.22 (61.82%)		18575892.98 (85.44%)	1900161.80 (48.81%)		16133790.36 (82.25%)	4342264.42 (72.16%)		18138218.40 (84.61%)	2337836.38 (55.72%)	
Non-Hispanic Black	1221535.80 (6.01%)	942140.03 (17.73%)		1294262.36 (5.95%)	869413.47 (22.33%)		1609621.42 (8.21%)	554054.41 (9.21%)		1360409.56 (6.35%)	803266.27 (19.15%)	

Other Race	758827.49 (3.73%)	395588.32 (7.45%)		933130.05 (4.29%)	221285.76 (5.68%)		776115.75 (3.96%)	378300.06 (6.29%)		912867.31 (4.26%)	241548.51 (5.76%)	
**Ratio of family income to poverty [2]**	3.17 (1.58)	2.27 (1.48)	<0.001	3.21 (1.55)	1.78 (1.28)	<0.001	3.16 (1.58)	2.43 (1.54)	<0.001	3.18 (1.56)	2.04 (1.46)	<0.001
(Missing)	1226083	544724		1504131	266676		1453393	317415		1533677	237131	
**Poverty–income ratio (%) [1]**			<0.001			<0.001			<0.001			<0.001
≤0.99	1483854.20 (7.77%)	969835.02 (20.34%)		1267264.02 (6.26%)	1186425.20 (32.72%)		1361531.65 (7.50%)	1092157.57 (19.16%)		1363234.56 (6.85%)	1090454.66 (27.55%)	
≥1	17610439.15 (92.23%)	3798616.29 (79.66%)		18969536.62 (93.74%)	2439518.82 (67.28%)		16800752.84 (92.50%)	4608302.60 (80.84%)		18540962.11 (93.15%)	2868093.33 (72.45%)	
(Missing)	1226083	544724		1504131	266676		1453393	317415		1533677	237131	
**Body mass index (%)[1]**			0.3			0.8			0.6			0.9
< 25 kg/m^2	5787415.75 (28.59%)	1460882.04 (28.06%)		6095775.92 (28.10%)	1152521.87 (30.63%)		5613706.13 (28.78%)	1634591.65 (27.50%)		6113208.62 (28.68%)	1135089.17 (27.46%)	
25-30 kg/m^2	6833059.60 (33.75%)	1473368.26 (28.30%)		7145749.84 (32.95%)	1160678.02 (30.85%)		6486627.83 (33.25%)	1819800.03 (30.62%)		6975457.31 (32.72%)	1330970.55 (32.20%)	
≥30 kg/m^2	7625840.09 (37.67%)	2271751.05 (43.64%)		8448292.15 (38.95%)	1449298.99 (38.52%)		7408139.00 (37.97%)	2489452.14 (41.88%)		8229648.24 (38.60%)	1667942.90 (40.35%)	
(Missing)	74061	107174		51114	130121		107204	74031		119559	61676	
**Body Mass Index (kg/m^2) [2]**	28.97 (6.44)	29.63 (7.09)	0.4	29.04 (6.47)	29.49 (7.17)	0.8	29.03 (6.65)	29.35 (6.35)	0.4	29.02 (6.53)	29.57 (6.84)	0.5
(Missing)	74061	107174		51114	130121		107204	74031		119559	61676	

**Diabetes (%)**			0.004			<0.001			<0.001			<0.001
Without Diabetes	16774330.48 (82.55%)	3756613.46 (70.81%)		17969603.04 (82.65%)	2561340.89 (65.93%)		16383738.41 (83.56%)	4147205.53 (68.91%)		17871106.22 (83.39%)	2659837.71 (63.39%)	
With Diabetes	3546046.24 (17.45%)	1548947.22 (29.19%)		3771328.97 (17.35%)	1323664.49 (34.07%)		3224324.23 (16.44%)	1870669.24 (31.09%)		3559152.49 (16.61%)	1535840.97 (36.61%)	
(Missing)	0	7614		0	7614		7614	0		7614	0	
**Had at least 12 alcohol drinks/year (%)** [1]			0.003			<0.001			0.015			<0.001
No	7005811.36 (34.97%)	2514912.33 (47.57%)		7387649.61 (34.31%)	2133074.08 (56.30%)		6811477.51 (35.17%)	2709246.17 (45.51%)		7329258.86 (34.57%)	2191464.83 (53.24%)	
Yes	13027277.16 (65.03%)	2771383.99 (52.43%)		14142974.52 (65.69%)	1655686.62 (43.70%)		12555029.44 (64.83%)	3243631.70 (54.49%)		13874072.79 (65.43%)	1924588.36 (46.76%)	
(Missing)	287288	26879		210308	103859		249170	64997		234542	79626	
**Hypertension (%)** [1]			<0.001			<0.001			0.3			<0.001
Without hypertension	8826155.83 (43.52%)	1587208.70 (29.93%)		9357312.94 (43.14%)	1056051.59 (27.13%)		8227340.27 (42.03%)	2186024.25 (36.39%)		9294951.26 (43.44%)	1118413.26 (26.72%)	
With hypertension	11454528.61 (56.48%)	3715751.03 (70.07%)		12333711.37 (56.86%)	2836568.28 (72.87%)		11348644.56 (57.97%)	3821635.08 (63.61%)		12103229.65 (56.56%)	3067049.99 (73.28%)	
(Missing)	39692	10215		49908	0		39692	10215		39692	10215	
**Material status (%)** [1]			0.006			<0.001			0.022			0.002

Widowed/divorced/separated/never married	8918967.65 (43.89%)	2845521.33 (53.63%)		9420321.52 (43.34%)	2344167.46 (60.22%)		8564993.11 (43.68%)	3199495.86 (53.17%)		9306923.37 (43.43%)	2457565.61 (58.57%)	
Married/living with partner	11401409.08 (56.11%)	2460711.87 (46.37%)		12313668.54 (56.66%)	1548452.40 (39.78%)		11043742.04 (56.32%)	2818378.90 (46.83%)		12124007.87 (56.57%)	1738113.08 (41.43%)	
(Missing)	0	6942		6942	0		6942	0		6942	0	
**Smoking status (%) [1]**			0.7			0.075			0.7			0.7
Never	12000513.80 (59.08%)	3314114.80 (62.38%)		12856356.84 (59.15%)	2458271.76 (63.15%)		11796854.26 (60.16%)	3517774.33 (58.46%)		12744622.72 (59.47%)	2570005.88 (61.25%)	
Former	6254705.57 (30.79%)	1527873.04 (28.76%)		6850740.74 (31.52%)	931837.87 (23.94%)		5970465.39 (30.45%)	1812113.22 (30.11%)		6613490.91 (30.86%)	1169087.70 (27.86%)	
Current	2057674.38 (10.13%)	471187.32 (8.87%)		2026351.46 (9.32%)	502510.24 (12.91%)		1840874.49 (9.39%)	687987.21 (11.43%)		2072276.59 (9.67%)	456585.11 (10.88%)	
(Missing)	7483	0		7483	0		7483	0		7483	0	
**Education level (%) [1]**			<0.001			<0.001			<0.001			<0.001
Below high school	2405039.19 (11.84%)	1681008.89 (31.64%)		2264850.54 (10.42%)	1821197.54 (46.79%)		2340030.08 (11.93%)	1746018.00 (29.01%)		2259469.35 (10.54%)	1826578.73 (43.53%)	
High school	4714639.64 (23.20%)	1418453.68 (26.70%)		5140075.52 (23.64%)	993017.80 (25.51%)		4537885.41 (23.13%)	1595207.92 (26.51%)		4918907.45 (22.94%)	1214185.88 (28.94%)	
Above high school	13200697.89 (64.96%)	2213712.59 (41.66%)		14336005.95 (65.94%)	1078404.52 (27.70%)		12737761.62 (64.94%)	2676648.86 (44.48%)		14259496.40 (66.52%)	1154914.08 (27.53%)	

**Total Cholesterol (mg/dL)** [2]	206.99 (40.44)	195.67 (42.45)	<0.001	206.39 (40.28)	194.88 (44.27)	<0.001	207.26 (40.52)	196.10 (41.91)	0.003	207.61 (40.50)	189.48 (40.90)	<0.001
**Total Cholesterol (%)** [1]			0.012			<0.001			0.003			<0.001
< 174 mg/dL	4096418.90 (20.16%)	1654111.38 (31.13%)		4449397.73 (20.47%)	1301132.55 (33.43%)		3898269.85 (19.87%)	1852260.43 (30.78%)		4232852.29 (19.74%)	1517677.99 (36.17%)	
174-201 mg/dL	5264620.88 (25.91%)	1378810.11 (25.95%)		5523581.22 (25.41%)	1119849.77 (28.77%)		4997238.82 (25.48%)	1646192.17 (27.36%)		5330610.85 (24.87%)	1312820.14 (31.29%)	
201-229 mg/dL	5303208.29 (26.10%)	1264566.79 (23.80%)		5961871.72 (27.42%)	605903.36 (15.57%)		5193694.01 (26.48%)	1374081.07 (22.83%)		5857104.41 (27.32%)	710670.67 (16.94%)	
>229 mg/dL	5656128.66 (27.83%)	1015686.87 (19.12%)		5806081.35 (26.71%)	865734.18 (22.24%)		5526474.43 (28.17%)	1145341.10 (19.03%)		6017305.65 (28.07%)	654509.88 (15.60%)	

CERAD(Consortium to Establish a Registry for Alzheimer's Disease); Data is number of subjects (percentage) or medians (interquartile ranges); [1] Chi-square test was used to compare the percentage between participants with and without low cognitive performance; [2] Wilcoxon rank sum test or Fisher's exact test was used to compare the mean$\pm$standard deviance values between participants with and without low cognitive performance.
