# Supplementary material for: Higher Total Cholesterol Concentration May Be Associated with Better Cognitive Performance among Elderly Females
Source: Nutrients. 2022 Oct 9;14(19):4198. doi: 10.3390/nu14194198 (PMC9571708; doi:10.3390/nu14194198)
Supplement: Supplementary file 1 [file nutrients-14-04198-s001.zip › Supplement Table S2.pdf]

**Supplement Table S2** Weighted odds ratios (95% confidence intervals) for four dimensions of cognitive performance across quartiles of total cholesterol in survey data

| | CFDAST_SCORE | | | CFDDS_SCORE | | | CDEAR_SCORE | | | z_score | | |
|---|---|---|---|---|---|---|---|---|---|---|---|---|
| | **Model-1** | **Model-2** | **Model-3** | **Model-1** | **Model-2** | **Model-3** | **Model-1** | **Model-2** | **Model-3** | **Model-1** | **Model-2** | **Model-3** |
| < 174 mg/dL | 1.00 (Ref.) | 1.00 (Ref.) | 1.00 (Ref.) | 1.00 (Ref.) | 1.00 (Ref.) | 1.00 (Ref.) | 1.00 (Ref.) | 1.00 (Ref.) | 1.00 (Ref.) | 1.00 (Ref.) | 1.00 (Ref.) | 1.00 (Ref.) |
| 174-201 mg/dL | 0.65 (95%CI 0.37-1.13) | 0.74 (95%CI 0.40-1.34) | 0.77 (95%CI 0.40-1.49) | 0.69 (95%CI 0.42-1.15) | 0.83 (95%CI 0.45-1.54) | 0.92 (95%CI 0.47-1.77) | 0.69* (95%CI 0.48-0.99) | 0.78 (95%CI 0.53-1.15) | 0.91 (95%CI 0.58-1.42) | 0.69 (95%CI 0.44-1.06) | 0.85 (95%CI 0.52-1.41) | 0.99 (95%CI 0.56-1.74) |
| 201-229 mg/dL | 0.59** (95%CI 0.40-0.87) | 0.82 (95%CI 0.53-1.25) | 0.90 (95%CI 0.55-1.46) | 0.35*** (95%CI 0.21-0.58) | 0.40** (95%CI 0.22-0.71) | 0.47* (95%CI 0.26-0.82) | 0.56* (95%CI 0.33-0.93) | 0.65 (95%CI 0.35-1.19) | 0.77 (95%CI 0.41-1.46) | 0.34*** (95%CI 0.22-0.51) | 0.38*** (95%CI 0.24-0.62) | 0.46** (95%CI 0.27-0.79) |
| >229 mg/dL | 0.44*** (95%CI 0.30-0.66) | 0.53** (95%CI 0.35-0.81) | 0.57* (95%CI 0.35-0.93) | 0.51** (95%CI 0.34-0.77) | 0.57* (95%CI 0.34-0.97) | 0.65 (95%CI 0.38-1.11) | 0.44*** (95%CI 0.28-0.68) | 0.47** (95%CI 0.28-0.78) | 0.55* (95%CI 0.32-0.95) | 0.30*** (95%CI 0.20-0.46) | 0.28*** (95%CI 0.15-0.52) | 0.34** (95%CI 0.17-0.65) |
| *P*-trend | <0.001 | <0.007 | <0.042 | <0.001 | <0.009 | <0.026 | <0.001 | <0.01 | <0.043 | <0.001 | <0.001 | <0.001 |

DDST(Digit Symbol Substitution Test); CERAD(Consortium to Establish a Registry for Alzheimer's Disease); Calculated using binary logistic regression; Reference (Ref.); Model 2 adjusted for age, education level, body mass index (BMI), marriage status, Ratio of family income to poverty and race; Model 3 adjusted for age and race, educational level, marriage status, Ratio of family income to poverty, body mass index (BMI), drinking status, smoking status, hypertension, and diabetes.* $P < 0.05$; ** $P < 0.01$; *** $P < 0.001$
