# Supplementary material for: Higher Total Cholesterol Concentration May Be Associated with Better Cognitive Performance among Elderly Females
Source: Nutrients. 2022 Oct 9;14(19):4198. doi: 10.3390/nu14194198 (PMC9571708; doi:10.3390/nu14194198)
Supplement: Supplementary file 1 [file nutrients-14-04198-s001.zip › Supplement Table S3_.pdf]

Supplement Table S3 Missing Data of Variables for table 2

Variables	Missing (%)
Body mass index	13 (0.99)
Diabetes	1 (0.076)
Had at least 12 alcohol drinks/year	17 (1.3)
Hypertension	2 (0.15)
Material status	1 (0.076)
Smoking status	1 (0.076)
